# Theoretical perspectives on the infectiousness of Ebola virus disease

**DOI:** 10.1186/1742-4682-12-1

**Published:** 2015-01-06

**Authors:** Hiroshi Nishiura, Gerardo Chowell

**Affiliations:** Graduate School of Medicine, The University of Tokyo, 7-3-1 Hongo, Bunkyo-ku, Tokyo, 113-0033 Japan; CREST, Japan Science and Technology Agency, Honcho 4-1-8, Kawaguchi, Saitama, 332-0012 Japan; School of Public Health, Georgia State University, Atlanta, GA USA; Division of International Epidemiology and Population Studies, Fogarty International Center, National Institutes of Health, Bethesda, MD 20892 USA; School of Human Evolution and Social Change, Arizona State University, Tempe, AZ 85282 USA

## Abstract

**Background:**

Ebola virus disease (EVD) has generated a large epidemic in West Africa since December 2013. This mini-review is aimed to clarify and illustrate different theoretical concepts of infectiousness in order to compare the infectiousness across different communicable diseases including EVD.

**Methods:**

We employed a transmission model that rests on the renewal process in order to clarify theoretical concepts on infectiousness, namely the basic reproduction number, *R*_0_, which measures the infectiousness per generation of cases, the force of infection (i.e. the hazard rate of infection), the intrinsic growth rate (i.e. infectiousness per unit time) and the per-contact probability of infection (i.e. infectiousness per effective contact).

**Results:**

Whereas *R*_0_ of EVD is similar to that of influenza, the growth rate (i.e. the measure of infectiousness per unit time) for EVD was shown to be comparatively lower than that for influenza. Moreover, EVD and influenza differ in mode of transmission whereby the probability of transmission per contact is lower for EVD compared to that of influenza.

**Conclusions:**

The slow spread of EVD associated with the need for physical contact with body fluids supports social distancing measures including contact tracing and case isolation. Descriptions and interpretations of different variables quantifying infectiousness need to be used clearly and objectively in the scientific community and for risk communication.

## Background

An epidemic of Ebola virus disease (EVD) centred in three West African countries has been ongoing since December 2013, with limited international spread to other countries in Africa, Europe and the USA [1]. It is likely that the duration of this EVD epidemic, associated with a high case fatality risk (CFR) estimated at ~70% [2, 3], will extend well into 2015. To investigate the ongoing EVD transmission dynamics and consider a range of possible countermeasures, it is vital to understand the natural history and epidemiological dynamics of this disease.

Owing to the rapid progression of the EVD epidemic in West Africa, attempts have been made to clarify the fundamental epidemiological characteristics of EVD [1, 2, 4]. For instance, several studies have reported statistical estimates of the reproduction number, i.e., the average number of secondary cases generated by a single primary case, as a measure of the transmission potential of EVD [2, 5–12]. Despite substantial progress, it remains unclear how measures of infectiousness (or the transmissibility) of EVD should be communicated to the public and interpreted in light of the set of control interventions that could be considered in practical settings. Hence, the purpose of this mini-review is to comprehensively classify different theoretical aspects of infectiousness using a basic transmission model formulated in terms of a renewal process. This approach allows us to compare different measures of infectiousness across different communicable diseases and design possible countermeasures.

## Discussion

### Renewal process

Here we briefly review the definition of the basic reproduction number, *R*_0_ using the renewal process model [13]. Let *i*(*t*) represent the incidence (i.e. the transient number of new cases) at calendar time *t*. Assuming that the contribution of initial cases to the dynamics is negligible, the renewal process is written as
1

where *A*(*s*) is the rate of secondary transmission per single primary case at its infection-age (i.e., the time since infection) *s*. Using *A*(*s*), one can model the dependency of the transmission dynamics on infection-age [14]. By far the most commonly used measure of infectiousness is the basic reproduction number, *R*_0_, which is computed as
2

and it can be interpreted as the number of secondary cases produced by a single primary case throughout its entire course of infection in a completely susceptible population. Although the concept of *R*_0_ is well-known, it is important to note from (2) that *R*_0_ results from the integration over all infection-ages. It is well known that the mathematical definition of *R*_0_ in a heterogeneously mixing population is described by using the multivariate version of (1) and the next-generation matrix that maps secondary transmissions between and within sub-populations. *R*_0_ is defined as the largest eigenvalue of this matrix [15, 16]. Similarly, the definition of *R*_0_ can be adapted to the situation of periodic infectious diseases by handling the seasonal dynamics using a vector and employing Floquet theory (see e.g., [17]).

Although *R*_0_ is clearly a dimensionless quantity, the conceptual interpretation from the renewal process (1) permits us to regard *R*_0_ as the average number of infected cases produced “per generation”. For this reason, *R*_0_ could also be referred to as the basic reproductive ratio, as it could be calculated as the ratio of secondary to primary cases.

Adopting the mass action principle of the so-called Kermack and McKendrick epidemic model, a non-linear version of the renewal equation (1) follows [13]:
3

where *s*(*t*) is the fraction susceptible at time *t*, *β*(*s*) the rate of transmission per single infected individual at infection-age *s*, and *Γ*(*s*) the survivorship of infectiousness at infection-age *s*. Here we define the force of infection, *λ*(*t*) as
4

which yields a measure of the risk of infection in a susceptible population. The force of infection can be interpreted as the hazard of infection in statistical sense -- the rate at which susceptible individuals are infected [18]. In the classical Kermack and McKendrick epidemic model, *λ*(*t*) is modelled as proportional to the disease prevalence [13]. The force of infection is useful for the analysis of incidence data.

### Comparison of three communicable diseases

Table 1 shows empirical estimates of *R*_0_ and the mean generation time for three different infectious diseases that are characterized by significantly different transmissibility and natural history parameters, i.e., measles, influenza H1N1-2009 and EVD [1, 19, 20]. The mean generation time, *T*_g_ can be mathematically derived from the transmission kernel in the renewal process (1), i.e.,

**Table 1:** **The basic reproduction number and mean generation time of three different diseases**

Disease	Basic reproduction number	Mean generation time (days) ^a^	Reference
Measles	15.0 (12–18)	12.0	[19]
Influenza (H1N1-2009)	1.4 (1.2-3.1)	2.8	[20]
Ebola virus disease	1.7 (1.5-2.0)	15.0 ^b^	[1]

^a^It should be noted that the mean generation time is shorter than the mean serial interval if there are asymptomatic transmissions [21].

^b^The mean incubation period of EVD is estimated to be 12 days [22] and 10 days [1], both shorter than the mean generation time.

5

The mode of transmission greatly differs for three diseases considered (Table 1). Measles is transmitted efficiently through the air while the transmission of influenza mostly occurs via droplet although airborne transmission is also possible in a confined setting [23]. In contrast, transmission of EVD is greatly constrained to physical contacts via body fluids [1]. Despite the differences in the mode of transmission for these diseases, it is important to note that the estimates of *R*_0_ for H1N1-2009 and EVD are not too different (Table 1). Does that indicate that influenza (H1N1-2009) and Ebola are similarly infectious?

While the average *R*_0_ for influenza and Ebola are similar, here we underscore that their underlying transmission dynamics show fundamental differences. This can be understood by analysing the intrinsic growth rate *r* for both diseases. Assuming that the early growth of each disease follows an exponential form, i.e., *i*(*t*) = *i*_0_exp(*rt*) (where *i*_0_ is a constant), the renewal equation (1) is rewritten as the so-called Euler-Lotka equation. Replacing *i*(*t*) in both sides of (1) by *i*_0_exp(*rt*) and cancelling exp(*rt*) from both sides, we obtain
6

yielding the relationship between *R*_0_ and the generation time,
7

where *g*(*s*) is the probability density function of the generation time. Equation (7) frequently appears in discussions of mathematical demography [24] and theoretical epidemiology [25], which is useful to describe how the relationship is determined between *R*_0_ and the intrinsic growth rate *r* as a function of the generation time distribution. For instance, if the generation time distribution follows the exponential distribution or Kronecker delta function, we obtain the well-known estimators of *R*_0_, i.e., *R*_0_ = 1 + *rT*_g_ and *R*_0_ = exp(*rT*_g_), respectively [26]. Assuming that *g*(*s*) follows a gamma distribution with the coefficient of variation *k*, we have
8

**Figure 1:**
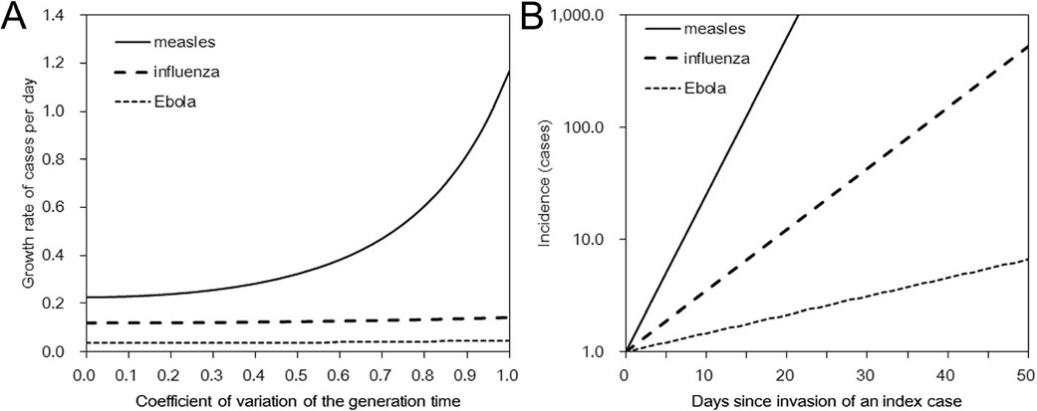
**Comparison of the intrinsic growth rate of infectious diseases. (A)** For a given *R*_0_ and the mean generation time *T*_g_ for a given infectious disease, the curves show the relationship between the intrinsic growth rate (*r*) and the coefficient of variation of the generation time of the disease. *r* of measles is the largest, followed by influenza, and then Ebola virus disease (EVD). **(B)** Temporal evolution in the number of new cases of measles, influenza, and Ebola virus disease using a coefficient of variation of the generation time at 50%. Parameter values are given in Table 1.

It should be noted that it is possible that the right-tail of *g*(*s*) for EVD might have been underestimated if there were substantial number of secondary transmissions from deceased persons during funerals. Adopting the values of *R*_0_ and *T*_g_ given in Table 1, and assuming that the coefficient of variation of the generation time at 50%, the intrinsic growth rate of influenza H1N1-2009 is calculated as 0.125 per day, while that of EVD is calculated as 0.038 per day. Figure 1A compares the growth rates (*r*) of three representative communicable diseases for different values of the coefficient of variation of the generation time. An epidemic of measles appears to grow the fastest followed by one of influenza while an outbreak of EVD is expected to grow the slowest. Whereas the *R*_0_ for EVD is similar to that of influenza, the growth rate of EVD is far smaller than that of influenza. This is because each disease generation in the context of EVD transmission takes approximately two weeks, while each generation of new influenza cases occurs on a much shorter time scale - every 3 days on average. Moreover, EVD spreads comparatively slowly mainly by physical contact. This feature indicates that social distancing measures including contact tracing and case isolation could be powerful options for controlling EVD assuming that public health infrastructure exists for these interventions to be feasible [27].

Thus, based on the infectiousness as measured by the growth rate of cases per unit time, it is very encouraging that EVD is far less dispersible than influenza. Although static countermeasures (e.g. mass vaccination at a certain age) can be planned using *R*_0_, the feasibility to deploy dynamic countermeasures, such as contact tracing and case isolation rests on the competition between the growth of cases and public health control, and in this context, the key parameter of infectiousness to assess the feasibility of control interventions is the intrinsic growth rate of cases.

### Per contact risk of infection

We further decompose the rate of secondary transmission per single primary case in the renewal equation (3) into the product of the contact rate *c*(*s*) and the per-contact probability of infection *p*(*s*), i.e.,
9

Assuming that the per contact probability of infection, *p* is independent of infection-age, we have
10

The interpretation of *p* is straightforward, i.e., it can be regarded as the risk of successful secondary transmission given an infectious contact to a susceptible individual. Assuming that everyone is susceptible at time zero, *R*_0_ in (2) is rewritten as
11

As mentioned above, *R*_0_ for EVD is similar to that of influenza. Nevertheless, the infectious period, modelled by *Γ*(*s*) for EVD is longer than that of influenza. Assuming an identical contact rate, *c,* between EVD and influenza, equation (11) indicates that the per-contact probability of infection for EVD is smaller than that for influenza.

**Figure 2:**
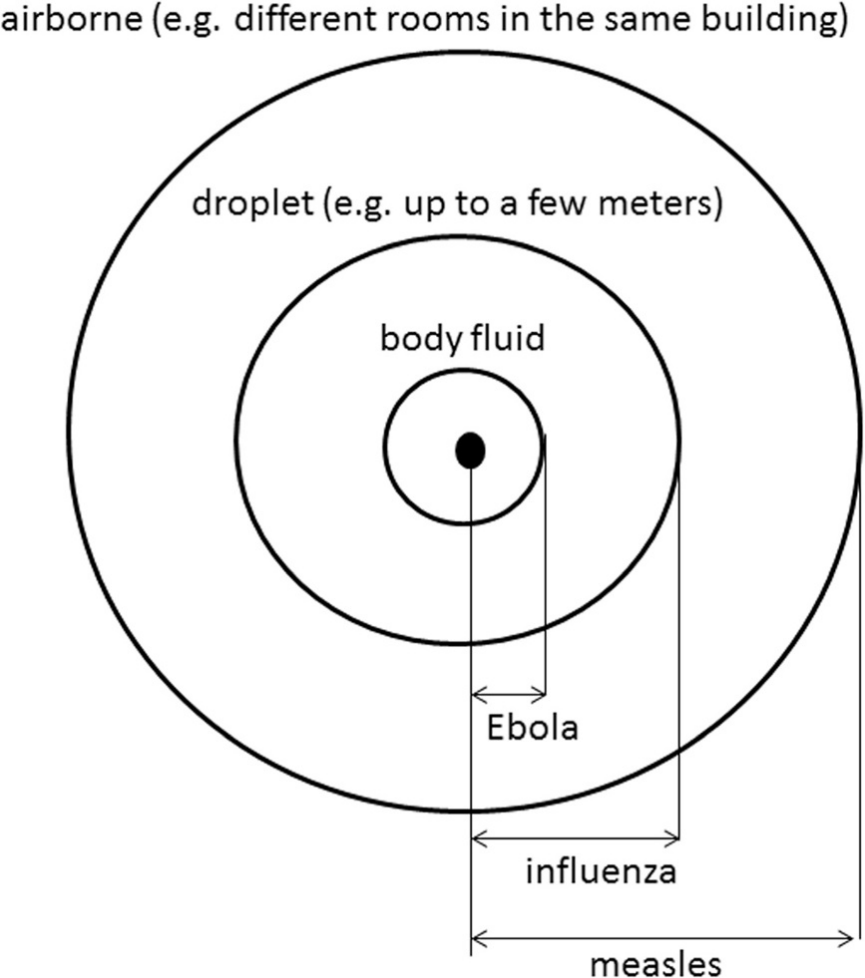
**The extent of the contact by different mode of transmission.** Airborne transmission can extend to different rooms and buildings, while the droplet transmission requires comparatively close contact. EVD is mainly transmitted through a physical exposure to body fluid of infected cases, and the extent of transmission is far less dispersible than those for measles and influenza.

The mode of transmission differs across communicable diseases. Figure 2 illustrates the physical range of “contact” that can potentially lead to infection for three representative infectious diseases. Measles causes airborne transmission, and thus, it can lead to secondary infections across different rooms (or sometimes even across buildings). The extent of contact for EVD is very limited as it is highly constrained to physical contacts with body fluids. Hence, effective contact for EVD is limited to close contacts that might be unavoidable among healthcare workers and household members of cases.

## Conclusion

We have comparatively discussed concepts of infectiousness for EVD in relation to other communicable diseases from a mathematical modelling point of view. The measure of infectiousness per generation of cases is *R*_0_. *R*_0_ offers a threshold principle and we have discussed that this measure is important for planning some static countermeasures such as mass vaccination. Based on *R*_0_, the overall infectiousness of EVD may be perceived to be similar to that of influenza. Nevertheless, the infectiousness per unit time for EVD was shown to be comparatively lower than influenza. The slow spread of EVD supports social distancing measures including contact tracing and case isolation. Moreover, the per-contact probability of infection for EVD is lower than that for influenza, and the mode of transmission also differs. These findings should also be regarded as encouraging news for healthcare workers who would have to have unavoidable and protected contact with EVD cases. In summary, there is a need for the use of clear and objective descriptions and interpretations of different variables quantifying infectiousness among the scientific community and for risk communication.

## Abbreviations

*R*_0_: The basic reproduction number
EVD: Ebola virus disease
CFR: Case fatality risk.
